# Spectrophotometric Determination of Metoprolol Tartrate in Pharmaceutical Dosage Forms on Complex Formation with Cu(II)

**DOI:** 10.3390/ph4070964

**Published:** 2011-06-28

**Authors:** Mustafa Cesme, Derya Tarinc, Aysegul Golcu

**Affiliations:** Department of Chemistry, Faculty of Science and Arts, University of Kahramanmaraş Sütçü Imam, Campuse of Avsar, 46100, Kahramanmaras, Turkey; E-Mail: mustafacesme@msn.com (M.C.)

**Keywords:** β-blockers, metoprolol, complexation, spectrophotometry

## Abstract

A new, simple, sensitive and accurate spectrophotometric method has been developed for the assay of metoprolol tartrate (MPT), which is based on the complexation of drug with copper(II) [Cu(II)] at pH 6.0, using Britton-Robinson buffer solution, to produce a blue adduct. The latter has a maximum absorbance at 675 nm and obeys Beer's law within the concentration range 8.5-70 μg/mL. Regression analysis of the calibration data showed a good correlation coefficient (r = 0.998) with a limit of detection of 5.56 μg/mL. The proposed procedure has been successfully applied to the determination of this drug in its tablets. In addition, the spectral data and stability constant for the binuclear copper(II) complex of MPT (Cu_2_MPT_2_Cl_2_) have been reported.

## 1. Introduction

**Figure 1:**
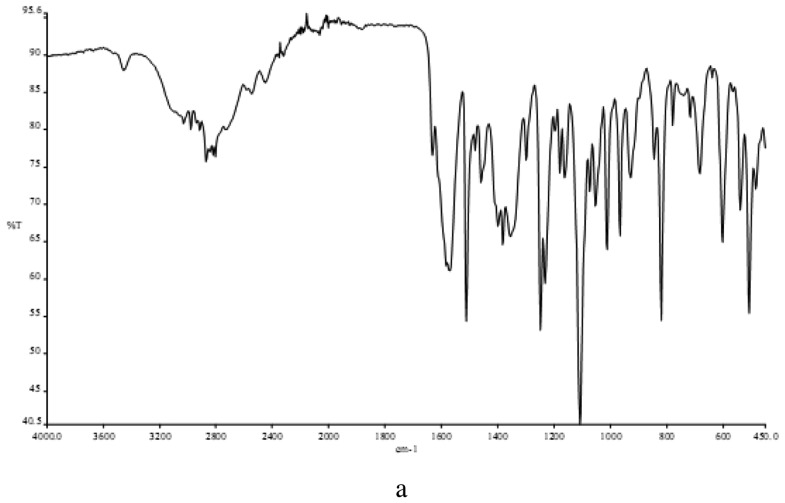

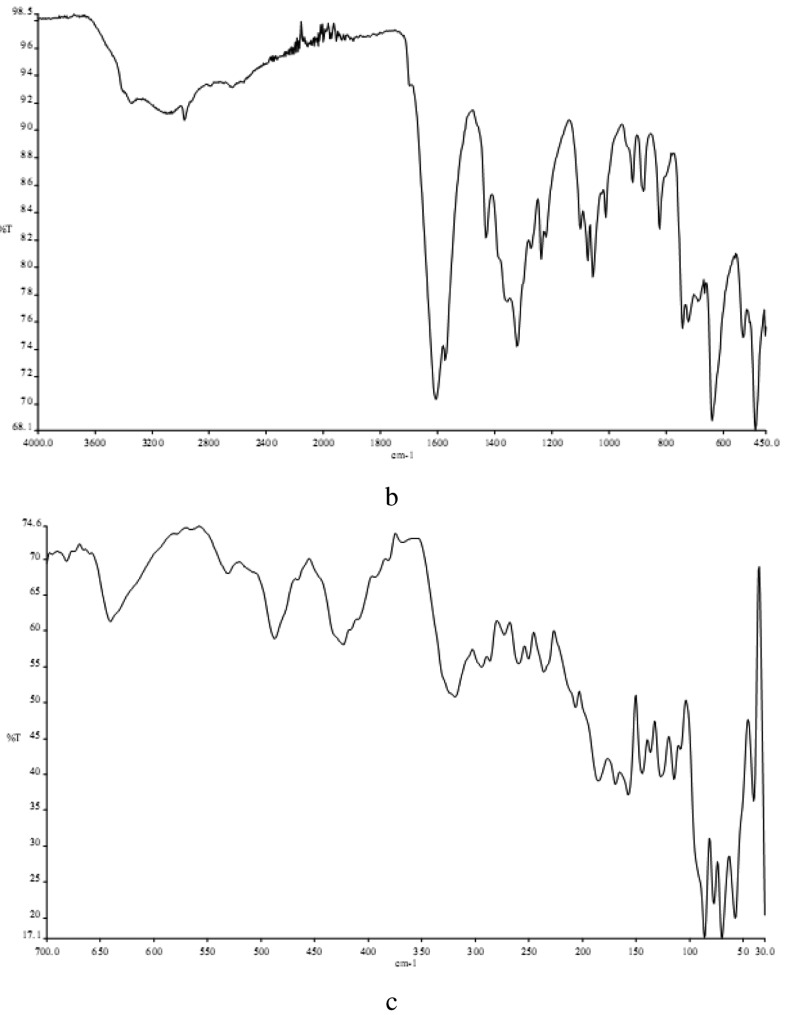
The infrared spectra of: **(a)** metoprolol tartrate; **(b)** binuclear MPT-Cu(II) complex; **(c)** far infrared of binuclear MPT-Cu(II) complex.

Metoprolol tartrate (Figure 1) is a selective β-adrenergic antagonist which is used in the treatment of cardiovascular disorders such as hypertension, angina pectoris, cardiac arrhythmias and myocardial infarction. The drug is quite sensitive, and even a small dose of the drug gives sufficient blockade. The β-blockers are also misused as doping agents in sports and therefore these drugs have been added to the list of forbidden drugs by the International Olympic Committee (IOC) [1]. Therefore the development of an analytical method for the determination of metoprolol tartrate is of great significance. The assay procedures of this drug listed in USP 23 NF18 (1995) [2] and BP (1998) [3] describe titrimetric and spectrophotometric methods [4,5]. In addition, several methods have been reported for quantification of MPT in plasma using high-performance liquid chromatography (HPLC) with UV or fluorescence detection [6-15]. Colorimetric determination of some β-blocking drugs using carbon disulphide and Cu(II) ions has been reported [16]. Chloroform was used as an extractant for the formed complex. Many of these methods involve a complex separation step and are non-reproducible. Although complexation reactions are very simple and sensitive, no method for the determination of MPT by this way has been reported. For many years, the use of complexation reactions as an analytical technique was used in lot of areas such as, paints and pigments, textile (mordantion reactions), dye-stuffs and determination of metals and drugs in a lot of materials (pharmaceuticals, biological samples, natural water samples, alloys and natural tea) [17-21]. In this paper, we present a spectrophotometric method for the determination of MPT based on its complexation reaction with Cu(II), which has then been applied to the analysis of MPT in tablets.

## 2. Experimental Section

### 2.1. Reagents

MPT and its dosage forms (Beloc Durules) were kindly provided by AstraZenaca Comp. (Istanbul, Turkey). Copper(II) chloride dihydrate and chemicals and solvents were obtained from E. Merck or Carlo Erba. Deionized water was used in order to ensure the absence of any undesirable ions.

### 2.2. Physical Measurements

The carbon, hydrogen and nitrogen analyses were obtained using a Leco 932 CHNS elemental analyser. The infrared spectra (4,000-400 cm^−1^) were obtained on a Perkin Elmer Spectrum 400 FTIR spectrophotometer using KBr discs. The electronic spectra were recorded in DMSO on a Perkin Elmer Lambda 45 spectrophotometer. Magnetic susceptibilities were measured by the Gouy method at room temperature using Hg[Co(SCN)_4_] as calibrant. Molar conductance of the complex was determined in water (∼10^−3^ M) at room temperature using a Jenway Model 4070 conductivity meter. The metal content of the complex was determined by an ATI Unicam 929 Model AA spectrometer in solutions prepared by decomposing the compounds in *aqua regia* and then subsequently digesting in concentrated HNO_3_/H_2_O_2_ (1/1).

### 2.3. Standard Solutions for Spectrophotometric Determination

A stock solution in water containing 0.2 mg/mL of MPT was prepared and was diluted as appropriate. This solution was stable for 1 week if kept in the refrigerator. CuCl_2_·2H_2_O solution 0.5% (w/v) was prepared in water.

### 2.4. Calibration Curve

Aliquot volumes of the stock solution containing 8.5-70 μg of MPT were transferred into a series of 10 mL volumetric flasks. Britton-Robinson buffer (1 mL) and CuCl_2_·2H_2_O solutions (1 mL) were added, mixed well for 20 min while heating using a thermostatically controlled water bath at 35 °C, and then cooled rapidly. The solutions were next completed to the mark with distilled water and the absorbance at 675 nm was measured against a reagent blank. A calibration curve was plotted and the corresponding regression equation then derived.

### 2.5. Procedure for Tablets

Ten tablets were weighed and pulverized, then a quantity of the powder equivalent to 40 mg MPT was transferred into a small conical flask and extracted with 4 × 20 mL of water, filtered into a 100 mL volumetric flask and completed to the mark with water. Aliquots were transferred into a 10 mL volumetric flask, than the procedure as described in Section 2.4 was applied. The nominal content of the tablet was determined either from the regression equation or using the calibration graph.

### 2.6. Preparation of the Binuclear Cu(II) Complex of MPT

**Scheme 2:**
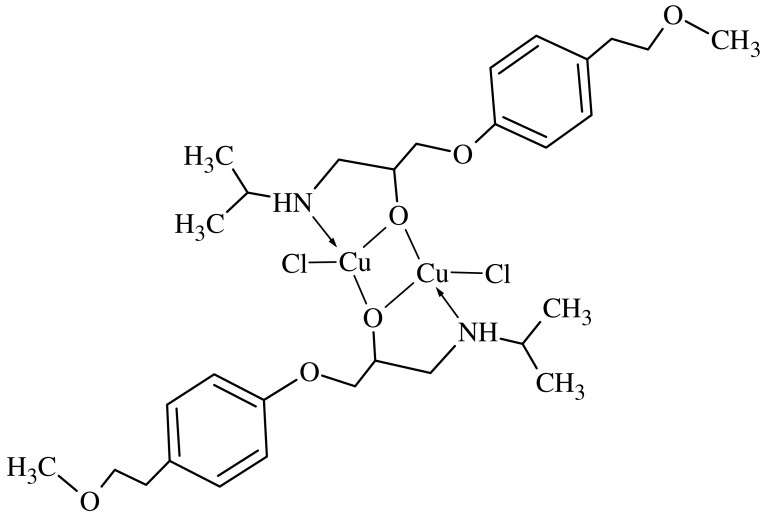
The structure of MPT_2_Cu_2_Cl_2_.

Methanolic CuCl_2_·H_2_O solution (20 mL, 0.171 g, 1.0 mmol) was added dropwise to a methanolic solution of MPT (20 mL, 0.267 g, 1.0 mmol) according to the literature [22]. The reaction mixture was heated using a thermostatically controlled water bath at 35 °C for 4 hours. The resulting blue precipitate was filtered, washed with methanol/water (1:1 v/v) and dried under vacuum over P_2_O_5_ for several days. Chloride ions in the resulting MPT_2_Cu_2_Cl_2_complex (Scheme 2) were determined by titration with AgNO_3_. Molecular weight: 730.71 gr/mol, Elemental analysis: (Found/Calculated): C%: 49.26/49.31, H%: 6.50/6.62, N%: 3.40/3.83, Cu%: 17.01/17.39, M.P. > 200 °C. Solubility: water, DMSO and acetonitrile/water (v/v, 1/1).

## 3. Results and Discussion

### 3.1. Characterization of the Binuclear Cu(II) Complex of MPT

The goal of this study was to synthesize a Cu(II) complex of the anthypertensive drug MPT, which is as a loop anthypertensive that blocks active sodium chloride transport in the thick ascending limb of Henle's loop. The [M]:[L] ratios were varied from 1:10 to 10:1 to find out the optimal ratio for complex formation. The effect of heat on the complex formation was investigated by changing the reaction temperature from +0 °C to the boiling point of the solvent used. The effect of pH on the complex formation was investigated; pH 6.0 was found to be the optimal value. The complex formation reactions were carried out with acetate and chloride salts of Cu(II). The highest yield for the MPT-metal complex was obtained with the chloride salt of Cu(II). This situation may be due to the role of the substituent groups attached to the benzene ring. Yield of the complex was investigated in different solvents such as water, methanol and acetonitrile. The best yield for the Cu(II) complex was obtained from water. The effect of the reaction time was also investigated. Reaction mixture was stirred in time intervals from 10 min to 48 hours. The molar conductance (Λ_M_) value (given in parentheses) in DMSO for MPT_2_Cu_2_Cl_2_ (21.42) suggests a 1:2 electrolytic complex [22].

### 3.2. Infrared Spectra

In determining the mode of binding of the ligand to the metal ion in the complex. The following three regions of the infrared spectrum have been studied:

(i) The 3459 cm^−1^ associated with the ν_(OH)_ vibrational mode.
(ii) The 2980 and 2872 cm^−1^ region associated with the ν_(NH2)_ and ν_(NH)_ modes.
(iv) The region 487-318 cm^−1^ due to ν_(M-N)_, ν_(M-O)_ and ν_(M-Cl)_ vibrations.

The spectra of the free ligand (MPT) showed absorption bands at 3459 and 2980-2872 cm^−1^ corresponding to the ν_(OH)_, ν_(NH2)_ and ν_(NH)_ stretching frequencies respectively, participating in the formation of intra- and/or intermolecular H-bonds. The binuclear Cu(II) complex of the MPT shows bands at 2977, 2892-3170 and 3213 cm^−1^, respectively, for ν_(NH2)_ and δ_(NH)_. No ν_(OH)_ bands were observed here because of the deprotonation of the alcohol oxygen. Sharp bands attributable to ν_(NH2)_ motion may be described as the asymmetric (1606 cm^−1^) N-H stretches, suggesting coordination with the NH group of the ligands (Figure 1) [23]. In the far-IR region, M-N, M-O and M-Cl vibrations were observed at 487, 430 and 318 cm^−1^, respectively, in accordance with literature data [24,25].

### 3.3. Electronic Absorption Spectra

**Figure 2:**
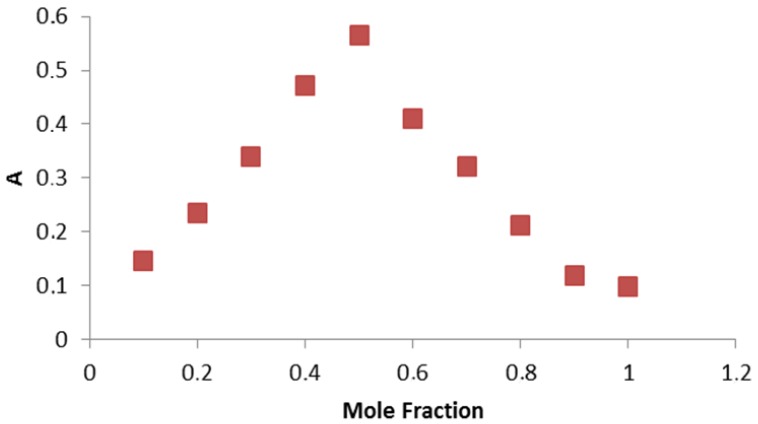
The continuous variation plot for the stoichiometry of the reaction of MPT and copper(II).

The electronic spectra of the complex was taken in water in order to obviate the effect of the solvent. The spectra of the complex contain some absorption bands in the 811-274 nm range (relatively weak, low-energy bands) which may be assigned to the d-d transitions in a square planar configuration. Thesse data are in accordance with the assumption for the formation of M-N, M-O and M-CI bonds [26]. The electronic spectra of the complex contain a band with a higher molar absorptivity in the 675 nm region, assigned to a primarily ligand-centered transition. The band at higher energy is associated with benzene π-π * transitions (261 nm). In order to study the stoichiometry of the coloured product the molar ratio between metal ions and drug was determined using Job's continuous variation method [22], which indicated a molar ratio of donor to acceptor of 1:1 for MPT with respect to the drug salt (Figure 2).

### 3.4. Atomic Absorption Spectra

The ratios of the metal present in the complex was determined by atomic absorption spectroscopy. The complex was decomposed in HNO_3_/H_2_O_2_ (1/1) and then dissolved in 1.5 N HNO_3_. The amount of Cu was determined. This supports the structure given in Scheme 2 [27-29].

### 3.5. Magnetic Measurement

The magnetic moment (B.M.) of the binuclear Cu(II) complex, (Cu(MPT)_2_(MeOH)_2_), was measured at room temperature found to be 1.25; this also supports the proposed structure [30,31]. The magnetic susceptibility of this complex obeys the Curie-Weiss law. Due to steric interactions of the larger size of the ligand the lower coordination number four has been assigned to these complexes [31,32]. The binuclear Cu(II) complex may thus have the usual tetrahedral structure.

### 3.6. Analytical Data

Copper(II) is a labelling reagent for primary and secondary amines. Several pharmaceutical compounds such as atenolol, acebutolol and propranolol have been determined through this approach [30]. Metoprolol tartrate is secondary aliphatic amino derivative, that was found to react with Cu(II) with the formation of the binuclear complex resulting in blue adduct. Under the described experimental conditions, the blue adduct has a characteristic absorption spectrum with a maximum absorption at 675 nm.

**Figure 3:**
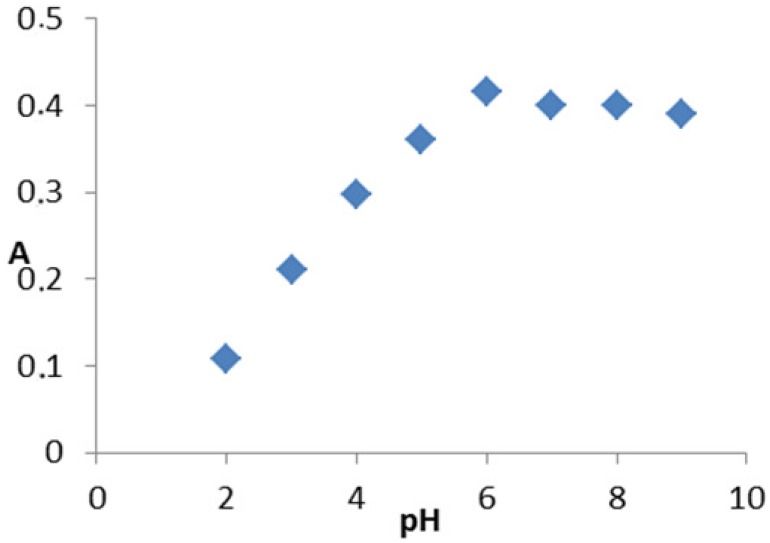
Effect of pH on the development of the complex of MPT (20 μg/mL) with copper(II).

The different experimental parameters affecting the produced color were extensively studied in order to determine the optimal conditions for the determination of the drug. First, the influence of pH on the absorption was studied. The maximum absorption using Britton-Robinson buffer occurs at approximately pH 6 (Figure 3). Other buffers such as carbonate or phosphate having the same pH value were studied and compared with Britton-Robinson buffer which was proven to be superior to the others because the absorbance readings were higher. This is due to the hydrolysis of Cu(II) to Cu(OH)_2_ using the other buffers. These results are in agreement with those of Miyano *et al* [33].

**Figure 4:**
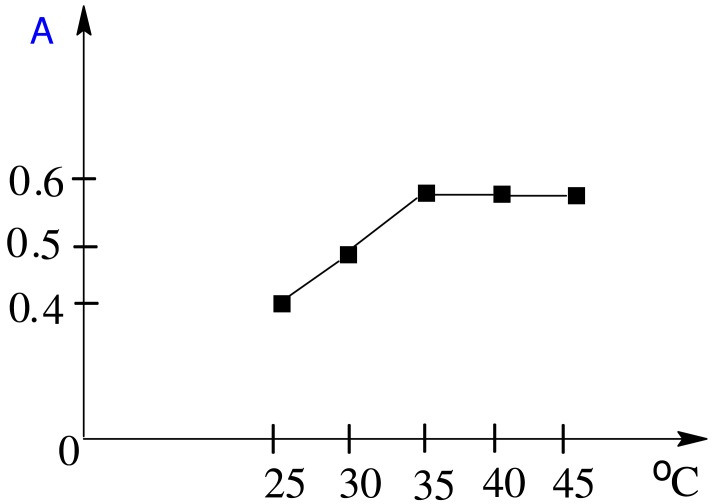
Effect of temperature on the development of the complex of MPT (20 μg/mL) with copper(II).

The effect of temperature on the produced adduct was studied, and it was found that heating at 35 °C for 20 min was better than heating at a higher temperature for a shorter period (Figure 4).

**Figure 5:**
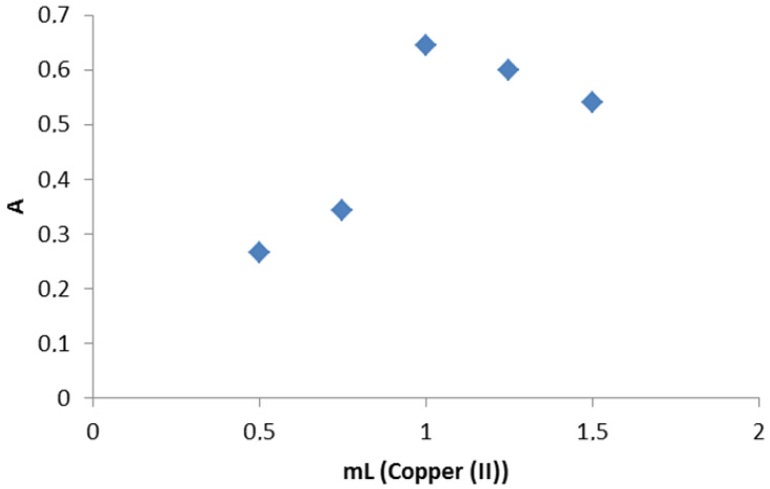
Effect of volume of copper(II) (0.5%, w/v) on the development of the complex of MPT (20 μg/mL) with copper(II).

The most important factor affecting the the produced blue color of the adduct was the volume of Cu^+2^. Figure 5 shows that 1 mL of 0.5% w/v Cu^+2^ solution gave maximum sensitivity. Increasing the volume of Cu^+2^ leads to a decrease in the absorbance, which may be due to the high background absorbance of the reagent. The absorption of the hydrolysis product of Cu^+2^, namely Cu(OH)_2_, completely disappeared at pH less than 4. Therefore, acidification of the reaction solution prior to the measurement remarkably decreased the background absorbancy without affecting the drug-metal adduct, hence, the sensitivity of the procedure increased.

Optimization of the reaction conditions. The spectrophotometric properties of the formed complex as well as the different experimental parameters affecting development and stability of the complex were carefully studied and optimized. For stability studies, the UV spectrophotometer was used. The absorbance values have been measured different times (5-60 min). The absorbance value is not change until 50 min. The absorbance value is decreased after 50 min (SD ± 0.03).

The stability constant of the reaction product *K*_f_ was calculated adopting the following formula [34]:

$$K_{\text{f}}=\frac{A/\text{Am}}{{\left(1-A/\text{Am}\right)}^{n+1}{\text{C}}^{n}{\text{n}}^{n}}$$

where *A* is maximum absorbance, Am is the absorbance corresponding to intersection of the two tangests of the curve in Figure 3, *C* is the concentration corresponding to maximum absorbance, *n* is the amount of the drug in reaction product. Using this equation, K*f* was found to be equal to 1.5 × 10^10^. Under the described experimental conditions, the relation between the absorbance at 675 nm with the concentration of MPT was linear over the 8.5-70 μg/mL range. Linear regression analysis of the concentration-absorption data gave the following equation:

A=70,000C−0.00271,r=0.998

where C is the concentration in μg/mL, A is the absorbance and ‘r’ is the correlation coefficient showing excellent linearity.

The limit of detection (LOD), expressed as the concentration at a signal-to-noise ratio 3 s/m, was 5.56 μg/mL and the limit of quantification (LOQ), expressed as the concentration at a signal-to-noise ratio 10 s/m, 7.11 μg/mL [31,32,35].

Statistical analysis of the results obtained by the proposed and official methods revealed no significant difference between the performance of the two methods regarding the accuracy and precision as shown in Table 1.

The proposed method was next applied to the determination of MPT in tablets. Inactive ingredients contained in the tablets are: sodium chloride and titanium dioxide. The results of analysis of tablets are shown in Table 2. Statistical analysis of the results obtained by both the proposed method and direct UV-spectrophotometric method shows no significant difference between the two methods regarding accuracy (*t*-test) and precision (*F*-test). The UV spectrophotometric method was validated by us and the results have been given in Table 2. However, the proposed procedure is advantageous over the reference methods since direct determination of Cu(II) can be done (if calibration curves of the metal are drawn). Also, the proposed method is sensitive, simple, and accurate. It can be applied for either content uniformity or routine quality control and no expensive laboratory techniques are needed.

### 4. Conclusions

A simple, sensitive and accurate spectrophotometric method has been developed for the assay of metoprolol tartrate (MPT), based on the complexation of drug with copper(II) [Cu(II)] at pH 6.0, using Britton-Robinson buffer solution, to produce a blue adduct. The developed method is more selective, sensitive, reproducible, rapid, cheap and simple than in most of the analogous methods cited in the literature [6-15]. For these reasons, it can be used in routine analysis and can be applied for the determination of MPT in pharmaceutical formulations and biological samples.

## Figures and Tables

**Scheme 1:**
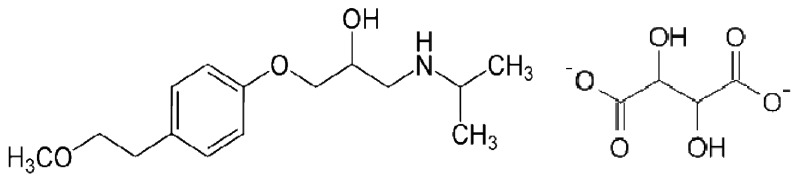
The structure of MPT.

**Table 1:** Regression data of the calibration line for quantitative determination MPT by UV method.

	Complexation method	UV-spectrophotometric method
Measured wavelength (λ_max as nm_)	675	274
Linearity range (μg/mL)	8.5-70	68.4-205.4
Slope	0.7 × 10^5^	0.25 × 10^4^
Absorbance range	0.254-0.921	0.276-0.79
ε*	91.900	2565
Intercept	-0.0271	0.0148
Correlation coefficient (r)	0.998	0.998
LOD (μg/mL)	5.56	8.10
LOQ (μg/mL)	7.11	26.98
Repeatability of absorbance (RSD%)	1.54	0.21
Repeatability of wavelength (RSD%)	0.04	0.02
Reproducibility of absorbance (RSD%)	0.45	0.63
Reproducibility of wavelength (RSD%)	0.12	0.03

* Molar absorption coefficients have been calculated for 30 μg/mL.

**Table 2:** Assay results from MPT dosage forms and mean recoveries.

	**MPT**
Product	Beloc Durules
Labeled claim (mg)	200
Amount found (mg) ^a^	199.50
RSD%	0.004
Bias%	−0.34
t-test (t_theoretical_ = 2.31)	0.70
F-test (F_theoretical_ = 2.60)	0.22
Added (mg)	40
Found (mg) ^a^	40.56
Recovery%	100.6
RSD% of recovery	1.30
Bias%	−0.46

a Each value is the mean of five experiments.
